# Clinical improvement despite rising anti-streptococcal antibody titers during adjunctive bacteriophage therapy in refractory PANDAS: a hypothesis-generating case report

**DOI:** 10.3389/fmed.2026.1973954

**Published:** 2026-10-19

**Authors:** Alfonso Recordare, Mariam Dadiani, Dea Nizharadze, Lia Nadareishvili, Giovanni Pirozzolo, Rubina Palumbo, Chiara Girotto, Giacomo Mezzapelle, Mzia Kutateladze

**Affiliations:** 1 Department of Surgery, Cittadella Hospital, ULSS6 Euganea, Padova, Italy; 2 Eliava Phage Therapy Center, Tbilisi, Georgia; 3 Unit of Microbiology and Virology, Schiavonia Hospital, ULSS6 Euganea, Padova, Italy; 4 George Eliava Institute of Bacteriophage Microbiology and Virology, Tbilisi, Georgia

**Keywords:** autoimmune neuropsychiatric disorder, bacteriophage therapy, molecular mimicry, neuroinflammation, PANDAS, pans, phage-antibiotic synergy, streptococcus pyogenes

## Abstract

**Background:**

Pediatric Autoimmune Neuropsychiatric Disorders Associated with Streptococcal infections (PANDAS) are characterized by the abrupt onset of anxiety, obsessive-compulsive symptoms, tics, and other neuropsychiatric manifestations associated with Group A Streptococcus exposure. Although antimicrobial and immunomodulatory therapies are widely employed, many patients experience persistent or relapsing symptoms despite prolonged treatment. Bacteriophage therapy has not previously been reported in PANDAS and represents an experimental, pathogen-directed therapeutic approach.

**Case presentation:**

We report the case of an 11-year-old girl with severe refractory PANDAS characterized by obsessive-compulsive symptoms, anxiety, motor and vocal tics, emotional dysregulation, insomnia, agitation, and marked functional impairment, including school refusal. The diagnosis was supported by elevated antistreptococcal serology. The patient received several escalating interventions, including intramuscular benzathine penicillin, azithromycin, plasma exchange, intravenous immunoglobulins, psychotherapy, and aripiprazole. A clinical improvement was initially observed after penicillin administration, although the benefit was incomplete and transient. No active streptococcal isolate was available for phage susceptibility testing. On the basis of repeated antibiotic responsiveness, empirical anti-streptococcal bacteriophage therapy was initiated using Pyo Bacteriophages (Eliava biopreparation), administered twice daily for three 15-day cycles as add-on therapy, while antibiotics were maintained.

**Results:**

Clinical sustained improvement became evident approximately six days after initiation of bacteriophage therapy, mirroring the latency previously observed after penicillin, and was followed by sustained clinical stabilization. No adverse events were reported. Serial serological evaluation demonstrated a paradoxical increase in ASO and anti-DNase titres despite continuous neurological and psychiatric improvement.

**Conclusions:**

This case suggests that bacteriophage therapy may represent a plausible pathogen-directed strategy in selected patients with refractory PANDAS, particularly when a reproducible clinical response to anti-streptococcal antibiotics suggests persistent microbial involvement. More importantly, the observed dissociation between rising anti-streptococcal antibody titers and sustained clinical improvement raises the possibility that serological kinetics may not accurately reflect disease activity during effective pathogen-directed therapy. One possible explanation is that bacteriophage-induced bacterial lysis increased antigen exposure while simultaneously reducing the bacterial burden responsible for persistent immune activation. However, because bacteriophages were administered as add-on therapy in a single uncontrolled case, causality cannot be established. Prospective studies are required to determine the role of bacteriophage-based interventions in post-infectious neuroimmune disorders.

## Introduction

Pediatric autoimmune neuropsychiatric disorders associated with streptococcal infections (PANDAS) were originally described as a subgroup of children with abrupt-onset obsessive-compulsive disorder and/or tic disorders temporally associated with Group A *β*-hemolytic Streptococcus (GAS) exposure (1–5). The proposed pathophysiology involves an aberrant immune response to streptococcal antigens, with molecular mimicry, anti-neuronal immune reactivity, basal ganglia dysfunction, and neuroinflammatory mechanisms considered biologically plausible contributors to symptom onset and recurrence (6–10).

Experimental studies have demonstrated that streptococcal-specific Th17 lymphocytes may promote blood–brain barrier dysfunction, facilitating the entry of immune mediators into the central nervous system and amplifying local inflammatory responses (11). Rather than representing a simple consequence of elevated anti-streptococcal antibody titers, disease activity is increasingly considered the result of a complex interaction among persistent antigenic stimulation, adaptive immune dysregulation, endothelial activation, and host genetic susceptibility. Consequently, circulating anti-streptococcal antibodies such as antistreptolysin O (ASO) and anti-DNase B are regarded as markers of previous streptococcal exposure rather than direct indicators of ongoing neuroimmune injury (8–12).

In clinical practice, confirming an active streptococcal reservoir at the time of neuropsychiatric exacerbation is often difficult. Patients may be evaluated after multiple antimicrobial courses, during symptom fluctuations, or in the absence of detectable pharyngeal colonization. Consequently, clinicians frequently rely on indirect evidence, including elevated anti-streptococcal antibody titers, temporal associations with infection, and clinical response to anti-streptococcal treatment.

Treatment strategies for PANDAS are commonly multimodal and may include symptomatic psychiatric management, cognitive-behavioral therapy, antimicrobial therapy when infection is suspected or documented, and immunomodulatory interventions in selected moderate-to-severe cases (3, 13–16). Plasma exchange and intravenous immunoglobulins have been used in severe infection-triggered obsessive-compulsive and tic disorders, although patient selection, timing, and durability of response remain clinically challenging (13–16).

Bacteriophages are naturally occurring viruses that specifically infect bacteria and have re-emerged as promising precision antimicrobial agents in response to increasing antimicrobial resistance. Beyond their direct bactericidal activity, experimental evidence suggests that bacteriophages may influence host immunity through interactions with innate and adaptive immune pathways, modulation of cytokine responses, and preservation of microbial ecological balance (17–25). Experimental and preclinical studies have further suggested that bacteriophages can translocate across epithelial and endothelial barriers, including, under specific conditions, the blood–brain barrier, raising the possibility that their biological effects may extend beyond simple bacterial eradication. Although the clinical significance of these observations remains uncertain, they provide a plausible mechanistic framework for investigating bacteriophages in neuroimmune disorders triggered by bacterial infections (26–29).

To our knowledge, bacteriophage therapy has not previously been reported in PANDAS. The present case describes empirical adjunctive bacteriophage treatment in a child with severe refractory disease whose therapeutic selection was guided not by microbiological isolation of *Streptococcus pyogenes* but by a reproducible clinical response to anti-streptococcal antibiotics. Beyond representing the first reported use of bacteriophages in this clinical setting, this case yielded an observation of particular mechanistic interest: sustained neurological improvement occurred despite a marked increase in ASO and anti-DNase B titers during therapy. This finding raises the hypothesis that anti-streptococcal antibody kinetics and disease activity may become dissociated during effective pathogen-directed treatment, possibly reflecting transient antigen release following bacteriophage-induced bacterial lysis rather than persistent pathogenic immune activation. Although this interpretation remains speculative, it may have important implications for the biological interpretation of serial anti-streptococcal serology and for current models of PANDAS pathogenesis (8–20).

## Case presentation

### Patient information

An 11-year-old girl presented with a severe relapsing neuropsychiatric syndrome characterized by anxiety, obsessive-compulsive symptoms, motor and vocal tics, emotional dysregulation, insomnia, episodic agitation, and marked impairment of daily functioning, including reduced school attendance and school refusal during exacerbations. Family history was notable for autoimmune disease in the maternal line, including connective tissue disease/spondyloarthritis. No psychiatric or autoimmune history was present in the paternal line. Developmental history was described as broadly unremarkable before the onset of the acute neuropsychiatric syndrome. The patient had previously shown good social functioning and school participation.

### Clinical history

The neuropsychiatric syndrome began abruptly in January 2024, with an episode of pharyngitis occurring in the patient's school class following a birthday party. Because the patient remained relatively asymptomatic at that time, no diagnostic microbiological testing or antimicrobial treatment was undertaken. In the same time 5 of the 17 children of the classroom developed pharyngitis, and were reportedly diagnosed with streptococcal infection, accordingly to information subsequently provided to the family by other parents. Approximately 15 days after this episode, the patient experienced an abrupt and dramatic neuropsychiatric episode. The parents reported that she did not recognize her mother and appeared markedly agitated and confused. At the same time, new motor phenomena emerged, including repetitive fluttering of the hands and arms, together with prominent anxiety. Within the following 2 days, she developed high fever (>39 °C), headache and neck stiffness, leading to hospital admission. During the hospital evaluation, the antistreptolysin O (ASO) title was >1:800. In view of the temporal relationship between the preceding pharyngeal illness, the acute neuropsychiatric onset, the subsequent febrile episode and the elevated streptococcal serology, the clinical picture was considered compatible with a post streptococcal neuroinflammatory syndrome, and treatment with penicillin was initiated. In spite of the clinical improvement with the antibiotic therapy, and the subsequent discharge from the hospital, neuropsychiatric symptoms progressed over several weeks, profoundly affecting family life, academic performance, sleep quality, and social interactions. The patient developed severe anxiety, obsessive thoughts and compulsive rituals, frequent motor and vocal tics, emotional dysregulation with episodes of aggression and crying, marked irritability, and progressive withdrawal from school and recreational activities.

Clinical evaluation performed in March 2024 was considered consistent with PANDAS according to contemporary diagnostic criteria. The clinical assessment also considered alternative explanations for the symptoms, including primary obsessive-compulsive disorder, tic disorder, anxiety-spectrum disorders and other neuropsychiatric conditions associated with abrupt symptom onset. The persistence of a temporal relationship with anti-streptococcal immune markers and the partial, reproducible response to anti-streptococcal treatment supported the clinical diagnosis of a post-streptococcal neuropsychiatric phenotype, although they did not establish a definitive causal relationship.

Neurological examination revealed no major focal deficits, although mild tandem gait instability was occasionally documented. The Children's Global Assessment Scale (CGAS) score was 31, indicating severe impairment in overall functioning. No standardized Yale Global Tic Severity Scale (YGTSS) and the Children's Yale-Brown Obsessive Compulsive Scale (CY-BOCS) assessment were available. Therefore, symptom severity and treatment response were evaluated through clinical observation, caregiver reports and functional outcomes.

### Diagnostic evaluation

The documented elevated anti-streptococcal markers, including antistreptolysin O titer of 1:800 and anti-DNase B of 460 U/mL. at the onset of the disease demonstrated evidence of recent streptococcal immune activation. No active Group A *Streptococcus* isolate could be recovered despite repeated microbiological investigations, preventing bacteriophage susceptibility testing and microbiological confirmation of persistent infection.

The absence of a positive throat culture was considered insufficient to exclude persistent antigenic stimulation, particularly because repeated antimicrobial exposure may reduce bacterial recovery while allowing bacterial persistence within biofilms, intracellular niches, or extra-pharyngeal reservoirs.

### Previous therapeutic interventions

During the following months, the patient underwent an extensive multidisciplinary therapeutic program. Oral penicillin initially produced clinically meaningful improvement, with reduction of anxiety, obsessive-compulsive symptoms, emotional dysregulation, and tic severity. However, the benefit consistently appeared approximately six days after treatment initiation and remained incomplete and transient.

Because of repeated relapses, long-term intramuscular benzathine penicillin prophylaxis was introduced between June 2024 and January 2025, again producing only partial clinical stabilization.

As disease severity increased, immunomodulatory therapy was progressively escalated. Seven sessions of therapeutic plasma exchange were performed, followed by three cycles of intravenous immunoglobulin (35 g administered over two consecutive days per cycle). Concomitant azithromycin therapy and low-dose aripiprazole were also prescribed to control persistent psychiatric symptoms. Despite these interventions, the patient continued to experience disabling neuropsychiatric manifestations with only limited and temporary benefit.

### Rationale for bacteriophage therapy

Repeated microbiological investigations did not provide an active streptococcal isolate suitable for pathogen-specific phage susceptibility testing. Therefore, the decision to initiate bacteriophage therapy was not based on direct microbiological confirmation or *in vitro* phage sensitivity. The therapeutic rationale was instead based on a reproducible clinical-biological signal: symptom improvement repeatedly occurred after anti-streptococcal penicillin administration, with a latency of approximately six days. This temporal pattern suggested that persistent or recurrent streptococcal involvement remained biologically plausible despite the absence of an identifiable bacterial isolate.

Given the refractory course, partial and transient response to antibiotics, limited benefit from immunomodulation, and the possibility of a persistent microbial trigger, empirical anti-streptococcal bacteriophage therapy was initiated.

### Bacteriophage intervention

Empirical bacteriophage therapy was performed using PYO Bacteriophages, a commercial available standardized phage polyvalent cocktail targeting *Streptococcus* spp. *Staphylococcus* spp. *Pseudomonas aeruginosa*, *Escherichia coli*, and *Proteus* spp. (Eliava biopreparation—Georgia) (30–32). Published descriptions of PYO Eliava biopreparation report an approximate overall phage concentration of 10^5^ plaque-forming Units (PFU)/mL for most components and at least 10^4 PFU/mL for the streptococcal component, although the exact composition and titre of individual phage components may vary. The preparation is commercially supplied in 10 mL vials. In the present case, PYO was administered orally, at a 10 mL dose, twice daily, for three cycles of 15 days. Because the treatment was experimental and because the patient had ongoing disease activity, bacteriophages were administered as add-on therapy while antimicrobial treatment with Penicillin was maintained. This point is critical for interpretation: the clinical response cannot be attributed to bacteriophage therapy alone, and a phage-antibiotic interaction or delayed effect of concomitant treatments cannot be excluded.

### Clinical outcome

Improvement became clinically evident approximately six days after initiation of bacteriophage therapy, remarkably reproducing the response latency previously observed after penicillin administration. During subsequent 6 weeks, progressive reduction of obsessive-compulsive symptoms, anxiety, emotional dysregulation, motor and vocal tics, sleep disturbance, and oppositional behavior was observed.

An additional indicator of clinical improvement was the patient's spontaneous acceptance of bacteriophage treatment after initially refusing therapy because of severe oppositional behavior, suggesting improved insight and treatment adherence as neuropsychiatric symptoms subsided.

Functional recovery was documented by substantial improvement in family interactions, school participation, peer relationships, and daily activities. Parent-reported Strengths and Difficulties Questionnaire (SDQ) Follow-up Impact Supplement scores (33, 34) decreased from 15/15 before bacteriophage therapy to approximately 6/15 following treatment, corresponding to an estimated 60% reduction in global symptom-related impairment. After completion of three bacteriophage cycles, the patient showed sustained clinical stabilization. Planned escalation of aripiprazole was no longer required according to the clinical timeline, and dosage was lowered in the last month. Functional recovery was reported during therapy. The patient also showed a clear improvement in her somatic and nutritional status. Her body weight increased from 29 kg before treatment to 32 kg during follow-up, while her height increased from 141 cm to 146 cm. Her appetite remained normal, and no enteral nutritional supplementation was required. This finding is particularly relevant in light of the patient's pre-treatment clinical condition, when her nutritional status had deteriorated to the point that enteral nutritional support through a feeding tube had been considered. No clinically significant adverse events, allergic reactions, gastrointestinal disturbances, or treatment discontinuations were observed during bacteriophage administration. The clinical course and key treatment milestones are summarized in Table 1.

**Table 1:** Timeline.

Date/period	Clinical event	Treatment	Reported response
January 2024	Abrupt severe neuroinflammatory syndrome with fever, anxiety, OCD symptoms, motor/vocal tics, emotional dysregulation, sleep disturbance.	Hospital admission and antibiotic treatment	Improvement followed by progressive deterioration
March 2024	Diagnosis consistent with PANDAS; ASO 1:800; anti-DNase B 460 U/mL	Oral penicillin	Improvement after approximately six days
June 2024–January 2025	Persistent relapsing disease activity	Repeated intramuscular benzathine penicillin	Partial and temporary benefit
February 2025	Severe refractory disease	Plasma exchange, seven sessions	Initial improvement reported
February 2025 onward	Adjunctive anti-infective and psychiatric treatment	Penicillin, azithromycin, aripiprazole	Incomplete response/partial stabilization
Late 2025/early 2026	Persistent disease activity	IVIG*, 35 g over two days; 3 cycles	Limited benefit
April 2026	Empirical anti-streptococcal bacteriophage therapy	Pyo Bacteriophages, twice daily, three 15-day cycles, (add-on therapy)	Improvement after approximately six days
June 2026	Completion of bacteriophage cycles	Continued bacteriophage and penicillin therapy	Sustained improvement
Follow-up September 2026	Clinical stabilization	Aripiprazole lowered; benzathin penicillin suspended	Functional recovery and somatic growth reported

*Intra Venous Immuno Globulins.

### Serological findings

One of the most unexpected observations concerned the evolution of anti-streptococcal serology. Rather than declining during clinical recovery, ASO titers, measured before phage therapy, increased from 170 IU/mL to 580 IU/mL after the third cycle, while anti-DNase B went up to 920 U/mL whereas neurological symptoms continued to improve, an observation that contrasts with the traditional interpretation of anti-streptococcal serology in PANDAS (6, 8, 10, 16).

This dissociation between increasing anti-streptococcal antibody titers and sustained clinical recovery constitutes one the hypothesis-generating observations of the present report and provided the basis for the mechanistic considerations discussed below.

## Discussion

This case describes empirical bacteriophage as additional therapy in a child with severe refractory PANDAS and highlights a potential translational principle: in selected post-streptococcal neuroimmune phenotypes, reproducible clinical responsiveness to anti-streptococcal antibiotics, even if transient and partial, may provide an indirect signal of persistent microbial involvement even when direct microbiological confirmation is not available.

The most relevant observation is the temporal concordance between the response to penicillin and the response to bacteriophage therapy. In both instances, improvement became apparent after approximately six days. This repeated latency does not prove a shared mechanism, but it supports the hypothesis that both interventions may have influenced a common pathogen-related process. In a condition such as PANDAS, where active infection is often difficult to demonstrate at the time of neuropsychiatric relapse, such response kinetics may be clinically informative. Although the clinical observation derives from a single uncontrolled case and therefore cannot establish causality, it raises several biologically relevant questions that extend beyond the potential therapeutic role of bacteriophages. In particular, the increase in anti-streptococcal antibody titers occurring simultaneously with sustained neurological improvement suggests that the relationship between humoral immune responses and disease activity in PANDAS may be more complex than is generally assumed.

The case also underscores the difficulty of distinguishing infection-driven immune activation from self-sustaining neuroinflammation. PANDAS is hypothesized to involve immune cross-reactivity between streptococcal antigens and neuronal targets, particularly within cortico-striato-thalamo-cortical circuits. Once initiated, neuroimmune activation may persist even in the absence of recoverable bacteria. Conversely, low-grade or anatomically inaccessible microbial reservoirs could theoretically maintain immune stimulation without being detected by routine testing. These possibilities are not mutually exclusive.

In this context, reproducible responsiveness to anti-streptococcal therapy may deserve investigation as a pragmatic surrogate biomarker for selecting patients who could benefit from pathogen-directed interventions.

### Bacteriophage therapy as precision antimicrobial medicine

Bacteriophages offer several theoretical advantages over conventional antibiotics. Their high specificity minimizes collateral disruption of the commensal microbiota, while their capacity to replicate selectively within susceptible bacterial populations may facilitate eradication of persistent bacterial reservoirs. Experimental evidence also suggests that bacteriophages may penetrate bacterial biofilms and act synergistically with antibiotics (35–38), potentially enhancing bacterial clearance in chronic infections (39–42).

Beyond their direct antibacterial activity, bacteriophages have increasingly been recognized as biological modulators capable of interacting with innate and adaptive immune responses. Experimental studies have demonstrated effects on macrophage activation, cytokine production, dendritic cell function, and mucosal immune homeostasis. These observations raise the possibility that clinical benefit may result not only from bacterial elimination but also from secondary modulation of immune pathways involved in chronic inflammation (17–23).

Although the relative contribution of these mechanisms cannot be determined in the present case, they provide a biologically plausible framework for investigating bacteriophage therapy in infection-triggered neuroimmune disorders such as PANDAS.

### Paradoxical increase in anti-streptococcal antibody titers

The most original finding of this report concerns the behavior of anti-streptococcal serology during treatment (6, 8, 10, 16). Rather than decreasing in parallel with clinical improvement, ASO titers increased from 170 to 580 IU/mL while anti-DNase B rose up to 920 U/mL. Importantly, this serological increase occurred without neurological deterioration and in parallel with progressive improvement of obsessive-compulsive symptoms, anxiety, emotional regulation, tic severity, and global functioning.

To our knowledge, this pattern has not previously been described during pathogen directed therapy in PANDAS.

One possible explanation is that the well known phenomenon of bacteriophage-induced bacterial lysis transiently increased the exposure of the immune system to streptococcal antigens. Enhanced antigen release could stimulate additional antibody production despite simultaneous reduction of the viable bacterial burden responsible for maintaining chronic immune activation (20, 22, 24). Under this hypothesis, increasing ASO concentrations would reflect an immunological response to antigen release rather than persistence or worsening of pathogenic disease activity (20–24).

Alternative explanations remain entirely possible. The observed antibody kinetics may simply reflect the natural evolution of humoral immunity, assay variability, or unrelated fluctuations in antibody production. Consequently, the present observation should not be interpreted as evidence of a causal relationship between bacteriophage-induced bacterial lysis and antibody dynamics. Rather, it should be regarded as a biologically plausible hypothesis requiring prospective validation.

Nevertheless, this observation raises an important conceptual question. Clinical practice often interprets increasing anti-streptococcal antibody titers as indirect evidence of persistent disease activity (6–12). However, experimental studies suggest that neurological injury in PANDAS is mediated through a far more complex interplay involving molecular mimicry, functional anti-neuronal antibodies, Th17 lymphocytes, endothelial activation, blood–brain barrier dysfunction, and local neuroinflammatory responses. Within this framework, circulating ASO and anti-DNase B concentrations may represent markers of immune exposure rather than direct indicators of ongoing pathogenic neuroimmune activity.

### Blood–brain barrier and neuroimmune implications

Growing experimental evidence indicates that communication between peripheral infection and the central nervous system involves dynamic interactions among adaptive immunity, endothelial integrity, and neuroinflammation. Streptococcal-specific Th17 lymphocytes have been shown to promote disruption of the blood–brain barrier, facilitating immune-cell trafficking into the central nervous system and amplifying inflammatory responses within basal ganglia circuits.

In parallel, experimental studies have suggested that bacteriophages may traverse epithelial and endothelial barriers, including, under selected experimental conditions, the blood–brain barrier. Although there is currently no evidence that this occurred in the present patient, these observations support the biological plausibility that bacteriophages may influence neuroimmune processes both indirectly through bacterial eradication and potentially through interactions with host immune pathways. Future mechanistic studies integrating microbiological analyses, immune profiling, neuroimaging, and pharmacokinetic evaluation of bacteriophages will be necessary to clarify these mechanisms.

### Future perspectives

Although this report cannot establish therapeutic efficacy, it identifies several priorities for future research. Prospective studies should evaluate serial ASO and anti-DNase B kinetics together with standardized neuropsychiatric outcome measures, immune-cell phenotyping, cytokine profiling, anti-neuronal autoantibodies, microbiome characterization, and microbiological investigation of potential streptococcal reservoirs. Controlled clinical trials will ultimately be required to determine whether bacteriophage therapy provides additional benefit beyond optimized antimicrobial and immunomodulatory treatment.

### Limitations

This report has several major limitations.

First, this is a single uncontrolled case. No causal inference can be made regarding bacteriophage efficacy.

Second, bacteriophages were administered as add-on therapy while antibiotics were maintained. Therefore, a phage-specific effect cannot be separated from phage-antibiotic synergy, delayed antibiotic effects, or combined treatment effects.

Third, no active streptococcal isolate was available, and no phage susceptibility assay was performed. The treatment was empirical rather than microbiologically personalized.

Fourth, the response assessment is based on clinical observation, caregiver report, and functional improvement. Future reports should include CY-BOCS, YGTSS, CGI-S/CGI-I, anxiety scales, sleep measures, and school-attendance metrics.

Fifth, the clinical course of PANDAS can be fluctuating, with spontaneous exacerbations and remissions. The temporal association between bacteriophage therapy and improvement is suggestive but not definitive.

Sixth, the patient received multiple prior and concomitant interventions, including antibiotics, plasma exchange, IVIG, psychotherapy, and aripiprazole. These treatments may have contributed to clinical stabilization.

## Conclusions

This report describes, to our knowledge, the first documented use of adjunctive bacteriophage therapy in a child with refractory PANDAS. Although no causal relationship between bacteriophage administration and clinical improvement can be established from a single uncontrolled observation, the case illustrates the potential value of pathogen-directed precision antimicrobial strategies in carefully selected patients who demonstrate reproducible responsiveness to anti-streptococcal therapy despite negative conventional microbiological investigations.

Beyond its therapeutic implications, this case raises a potentially important biological question. Sustained neurological and psychiatric improvement occurred despite a marked increase in anti-streptococcal antibody titers, suggesting that humoral immune responses and clinical disease activity may become dissociated during effective pathogen-directed treatment. One possible explanation, that remains unproven in this specific case, is that bacteriophage-mediated bacterial lysis may have transiently increased exposure to the immune system to streptococcal antigens. In fact, bacteriophage interactions with the host immune system and immune responses associated with bacterial lysis are very well clinically and experimentally documented. In this context, rising anti-streptococcal antibody titers during treatment would reflect increased antigen exposure rather than ongoing disease activity. Although entirely speculative, this hypothesis is biologically plausible and readily testable in prospective clinical studies.

The observed clinical improvement after bacteriophage therapy, the similar response latency to penicillin, and the absence of documented adverse events support further systematic investigation. However, because this was a single uncontrolled add-on intervention without microbiological confirmation, the findings must be interpreted cautiously. Prospective studies are needed to define patient selection criteria, safety, microbiological targets, immune correlates, and objective clinical outcomes.

From a neuroimmunological perspective, the case is relevant because it links a psychiatric-neurological phenotype, anti-streptococcal immune markers, antimicrobial responsiveness, immunomodulatory treatment, and a pathogen-directed experimental intervention. The central hypothesis is not that bacteriophages directly treat OCD or tics, but that they may reduce a microbial driver contributing to immune-mediated dysfunction in susceptible patients. This distinction is essential to avoid overinterpretation.

The implications are hypothesis-generating. Future studies should investigate whether patients with PANDAS who show reproducible antibiotic responsiveness differ immunologically from non-responders, whether they are more likely to benefit from pathogen-directed interventions, and whether bacteriophage therapy can be safely and rationally integrated with antimicrobial and immunomodulatory strategies.

## Data Availability

The raw data supporting the conclusions of this article will be made available by the authors, without undue reservation.
